# Minimal Clinically Important Differences for a Weight Distribution Platform in Dogs with Osteoarthritis

**DOI:** 10.3390/ani14010128

**Published:** 2023-12-29

**Authors:** J. C. Alves, Ana Santos, Catarina Lavrador, L. Miguel Carreira

**Affiliations:** 1 Divisão de Medicina Veterinária, Guarda Nacional Republicana (GNR), Rua Presidente Arriaga, 9, 1200-771 Lisbon, Portugal; 2 Faculty of Veterinary Medicine, Lusófona University, 1749-024 Lisbon, Portugal; 3 Centro de Ciência Animal e Veterinária, Lusófona University, 1749-024 Lisbon, Portugal; 4 MED—Mediterranean Institute for Agriculture, Environment and Development, Instituto de Investigação e Formação Avançada, Universidade de Évora, Pólo da Mitra, Ap. 94, 7006-554 Évora, Portugal; 5 Faculty of Veterinary Medicine, University of Lisbon (FMV/ULisboa), 1300-477 Lisbon, Portugal; 6 Interdisciplinary Centre for Research in Animal Health (CIISA), University of Lisbon, (FMV/ULisboa), 1649-004 Lisbon, Portugal; 7 Anjos of Assis Veterinary Medicine Centre (CMVAA), 2830-077 Barreiro, Portugal

**Keywords:** dog, chronic pain, osteoarthritis, orthopedics, minimal clinically important difference, weight distribution platform, objective assessment, stance analysis

## Abstract

**Simple Summary:**

Osteoarthritis is a very common joint disease in dogs and clinicians need reliable evaluation modalities to evaluate patients and monitor responses to treatment. Weight bearing evaluation platforms are objectives measures that are becoming more readily available in clinical settings. We aimed to evaluate what constitutes the minimal change that represents a clinical improvement with a weight bearing evaluation platform using different methodologies. We presented estimates that can be used for research and patient monitoring.

**Abstract:**

In this retrospective study to determine the optimal method of evaluating static weight-bearing distribution to assess response to treatment in dogs with osteoarthritis using a weight distribution platform, data from the Clinica Veterinária de Cães (Portuguese Republican National Guard) clinical records were extracted. At baseline and at 15 days post-treatment, follow-up data from 80 dogs treated for bilateral hip osteoarthritis were categorized based on an anchor question. Estimates of minimal clinically important differences were calculated with distribution-based and anchor-based methods for deviation from normal weight-bearing and a symmetry index (SI). For deviation, the anchor-based methods provided a range from −0.3 to −3.1, and the distribution-based methods from 0.16 to 0.29. For SI, the anchor-based methods provided a range from −10.0 to −23.9, and the distribution-based methods from 1.31 to 2.88. Receiver operator characteristic curves provided areas under the curve >0.7, indicating an acceptable cut-off point. We presented estimates of −1 for deviation and −10 for SI in dogs with OA. These estimates can be used for research and patient monitoring. Future studies should include OA from other joints and animals from a broader clinical context.

## 1. Introduction

Osteoarthritis (OA) represents at least 80% of the lameness and joint disease cases in companion animals. OA is affecting an expected growing number of animals worldwide, due to an increase in obesity rates and life expectancy [1,2]. A recent report looking at the prevalence of OA and associated clinical signs in young (aged 8 months to 4 years old) dogs found that 39.8% of animals had radiographic signs of OA in at least one joint. Also, 23.6% of dogs had clinical signs of OA, corresponding to an overlap of radiographic OA and joint pain in the same joint [3]. This means that the actual prevalence of OA may be underrepresented in available reports. Since osteoarthritis is a chronic progressive condition, clinicians should be aware of this reality to be able to evaluate each individual patient and detect OA early on, when a better response to treatment is expected [4].

For diagnosing and evaluating response to treatment, objective lameness assessment is an important tool [5]. Subtle changes in posture or weight-bearing may occur in the early stages of the disease process, which can be easily missed with visual assessment [6,7]. Weight distribution and off-loading or limb favoring at the stance are commonly used subjective assessments during the orthopedic examination, yet lameness may be difficult to detect during gait evaluation [8,9]. OA patients, in particular, may not be overtly lame at a walk or a trot but exhibit subtle shifts in body weight distribution at a stance due to pain or instability [5,10]. Stance analysis has been reported as a sensitive method for detecting dog lameness [11].

Weight distribution platforms have been proven to be repeatable and accessible devices to measure static weight distribution [12]. Stance analysis has been reported as sensitive for detecting lameness in dogs, compared to a pressure-sensitive walkway [5], allowing repeatable evaluations for dogs with hind limb lameness in single-day and multiday trials [13] and in the assessment of response to treatment [14]. Compared to a standard force plate, a stance analyzer does not require as much skill for data acquisition [15]. Also, in some conditions like OA, patients may not be able to undergo as many trials at the required velocity to obtain valid trials on a force plate. Still, they may be able to stand long enough to collect data using a stance analyzer [16]. Recently, their use has been reported in patients’ orthopedic evaluation, selecting the best candidates for certain surgeries, and assessing response to treatment [17,18].

The traditional assessment approach of determining whether any treatment or intervention had a measurable effect is to compare the overall mean or median differences in the evaluation modality performed between groups. An alternative approach is to predefine the criteria for the successful treatment of an individual patient so that the effect of the treatment or intervention in each animal can be determined [19]. In addition, there has been a recent increased focus over the last few years to define the change in a measurement that signifies an important improvement at the individual patient level [20]. This interest is based on the realization that a better result with a specific measure (e.g., a 1% symmetry index improvement) does not necessarily correspond to an important clinical improvement. For those reasons, specific minimal clinically important differences (MCID) must be determined for individual evaluation modalities.

This study aimed to estimate MCID for a weight distribution platform in dogs with hip OA.

## 2. Materials and Methods

This study is a part of a project approved by the ethical review committee of the University of Évora (Organismo Responsável pelo Bem-estar dos Animais da Universidade de Évora, approval no. GD/11670/2020/P1). Written informed consent was obtained from the institution responsible for the animals. Data were obtained from clinical information of dogs presented for treatment for bilateral hip OA at the Clinica Veterinária de Cães (Portuguese Republican National Guard). As part of the standard follow-up for these police working dogs, weight-bearing distribution is evaluated before treatment and at set intervals. The canine handlers complete copies of client-reported outcome measures, including an anchor question, the quality of life question of the Canine Brief Pain Inventory [21]. The only cases included in this study were patients that received a single treatment modality for both hips (e.g., intra-articular platelet-rich plasma administration or photobiomodulation). To be included in the present analysis, only animals that had not received any medication or nutritional supplements for over 6 weeks before the treatment and during the follow-up period and had no other joints affected were considered. Some of the information presented in the present study was collected during a different project but has not been reported before [22,23,24,25,26]. In this study, we followed a similar methodology outlined in published papers, looking at determining minimal clinically important differences for two client-reported outcomes measures (the Liverpool Osteoarthritis in Dogs—LOAD—and the Canine Orthopedic Index—COI), in dogs with osteoarthritis and following surgery for cranial cruciate ligament rupture [27,28]. For included animals, an anchor question was collected. This question corresponds to the global quality of life question is included at the end of the Canine Brief Pain Inventory questionnaire, aiming to obtain an owner’s overall assessment of their dog’s status [29]. This item was used as a criterion validity assessment in the validation of the severity and interference scores, the two dimensions of the Canine Brief Pain Inventory [29]. This question was “How do you describe your dog’s overall quality of life”. The possible responses were “Poor”, “Fair”, “Good”, “Very Good”, and “Excellent”. For the present study, data from the pre-treatment (T0) evaluation and the 15 days post-treatment follow-up (+15 d) were used. To be included, data from the patient at T0, +15 d, and anchor question answers had to be available.

The weight-bearing evaluation was conducted with a weight distribution platform (Companion Stance Analyser; LiteCure LLC^®^, Newark, DE, USA). The procedure of weight-bearing assessment has been described before [14]. Briefly, the equipment was placed in the center of a room, at least 1 m from the walls. The equipment was set to zero before each evaluation session. Animals were encouraged to stand on the platform, ensuring that one foot was placed on each quadrant. When required, gentle restraint could be used to keep the patient’s head facing forward. As observed in the analysis software, the animal’s center of gravity and stability should be near the platform’s middle point. Twenty measurements were obtained for each patient to determine a mean value. Two calculations were performed with the results of the weight-bearing evaluation: A deviation from the normal weight distribution for pelvic limbs was calculated by subtracting the weight-bearing of the limb from the value considered normal, i.e., 20% [5]. And a left–right symmetry index (SI) was also calculated with the formula: SI = [(WBR − WBL)/((WBR + WBL) × 0.5)] × 100, where WBR is the weight-bearing for the right limb and WBL is the weight-bearing for the left limb [30,31]. Negative values were made positive. All measurements were performed by the same clinician, blinded to the assigned group.

After the selection of patients, relevant clinical data were exported to an Excel (Microsoft, Seattle, WA, USA) spreadsheet. Subsequent statistical analyses were performed with commercially available software (IBM SPSS Statistics version 20—IBM, Armonk, NY, USA). Based on the canine handler’s responses to the anchor question at +15 d, two groups were set for further analyses. The first group, i.e., “the same” group, comprised the animals with the same responses at the T0 and +15 d evaluations, and the “somewhat better” group encompassed animals where a one-level better response was obtained at the +15 d evaluation compared to T0. The characteristics of the two groups at T0 were compared using the Mann–Whitney U test, while categorical data were compared using Fischer’s exact test. The Wilcoxon signed-rank test was used to compare changes in scores from T0 to +15 d. The Mann–Whitney U test was used to compare differences in the deviation and weight bearing and the mean change in the deviation and weight bearing between the two groups. A significance level was set at *p* < 0.05.

Following a similar methodology described previously [22], four different anchor-based approaches were used to calculate the MCID. The first was a determination of an “average change” (AC), which corresponds to the mean change in the score of the set “somewhat better” group. A “change difference” was also calculated. The “change difference” corresponds to the difference in the average change in score between the “somewhat better” and the “the same” groups. The “minimum detectable change” (MDC) was calculated, consisting of the smallest change that can be considered beyond the measurement error at a 95% confidence level. For deviation and SI, an improvement consists of a reduction in value. For that reason, the MCID was equal to the lower value of the 95% confidence interval for the average change in the “the same” group’s score. The fourth method was the receiver operating characteristic (ROC) curve. The ROC curve was used to define the point that best discriminated between the two groups. The point that maximized specificity and sensitivity was used to estimate this optimal cut-off point. We also calculated the area under the ROC curve (AUC). Based on available reports, AUC values between 0.7 and 0.8 were considered to have acceptable reliability and 0.8 and 0.9 were considered to have excellent reliability [28].

Two distribution methods were also used to estimate the MCID. First, the effect size was determined. The effect size was obtained from the difference in mean score from T0 to +15 d (for the present methodology) divided by the standard deviation (SD) of the T0 scores. MCID was calculated with the formula (SDT0 × 0.2), as an effect size of 0.2 is considered small [22]. A second method was used, the “standard error of measurement” (SEM), as SEM is an intrinsic property of the instrument considered and is independent of the patient cohort considered [22]. The formula SEM = SD × √(1 − r) was used, where “r” is the instrument’s reliability. A previous value has been reported for the weight-bearing platform [12].

## 3. Results

Data from 80 animals fulfilled the inclusion criteria, accounting for 160 limbs. Forty-eighty were intact males, and thirty-two were intact females, with a mean age of 6.9 ± 2.3 years and a body weight of 26.5 ± 4.9 kg. The breeds represented were German Shepherd dogs (*n* = 28), Belgian Malinois shepherd dogs (*n* = 24), Labrador Retrievers (*n* = 16), and Dutch Shepherd dogs (*n* = 12). Different treatments were identified: intra-articular hyaluronan (*n* = 20 limbs), intra-articular triamcinolone (*n* = 20 limbs), intra-articular stanozolol (*n* = 20 limbs), intra-articular platelet-rich plasma (*n* = 80 limbs), and photobiomodulation (*n* = 20 limbs).

The mean T0 and +15 d scores for deviation and SI are presented in Table 1. Both demonstrated a significant difference between pre-treatment and post-treatment scores.

Regarding the anchor question, 100 (62.5%) limbs were considered in the “somewhat better” and 80 (37.5%) were in the “the same” group. The scores at T0 and +15 d for these two groups are presented in Table 2. The MCID estimates with the different considered methods are shown in Table 3.

The four anchor-based methods provided a range of MCIDs for each measurement (0.3 to 3.1 for deviation and 10.0 to 23.9 for symmetry index). With the distribution-based methods, the MCID for deviation ranged from 0.16 (SEM) to 0.29 (effect size), while the MCID for symmetry index ranged from 1.31 (SEM) to 2.88 (effect size). Overall, a variation depending on the method applied was observed. Both AUCs calculated using the ROC curve were found to be acceptable (>0.7), with the highest for deviation (0.760) and the smallest for SI (0.729). This indicates an acceptable cut-off point.

## 4. Discussion

In the presented study, we estimated MCDIs for deviation and SI, obtained using a weight-bearing evaluation platform, in dogs with hip OA. This evaluation was performed with clinical data available from a clinical setting caring for working police dogs, submitted to different treatments. As for referenced studies looking at determining MCIDs for other clinical outcome instruments [27,28], although we selected a +15 d follow-up moment, this specific follow-up time-point should not be considered a recommendation for post-treatment evaluation in dogs with OA. Rather, it was a time-point chosen based on the previous experience of the clinicians, previous conducted research, and expectation that it would be enough time to obtain a response to treatment and therefore produce different answers to the anchor question, allowing us to estimate the MCID.

Static weight-bearing distribution has been used to evaluate patients after a certain procedure and detect differences between treatment and control dogs in several other reports [12,16,17,18]. Specifically for hip OA, static weight-bearing evaluation has been proposed to be equivalent or superior in evaluating pain to vertical impulse and peak vertical force [5,23]. Previous studies have shown the efficacy of the selected treatments in dogs with hip OA [24,26,31,32]. In this study, we provided MCID results in the clinical context of OA. Still, we need to remember that the estimates of MCIDs can be affected by extrinsic and intrinsic factors [33], and the dogs included comprise a very homogenous sample, representing specific breeds and similar housing, feeding, and exercise conditions. For those reasons, future studies are required to evaluate the proposed MCID in samples with different extrinsic and intrinsic factors.

We used different methods to generate the MCID estimates. While anchor-based estimates may be more clinically relevant, distribution methods are based on larger data sets [34]. Our four anchor-based methods have been used previously in human [35] and animal patients [36] and can generate different MCID estimates. The largest estimate for deviation was determined with “average change” at −3.1, and at −23.9 for the SI. Conversely, the lowest estimates were obtained with “standard error of measurement”, at −0.16 for deviation and −1.31 for SI. The AUC of the ROC curve showed an acceptable ability of deviation and SI to discriminate between the two groups of dogs. Considering these results, we propose a working MCID of −1 for deviation and −10 for SI for dogs with OA. This value is supported by the ROC and the “minimum detectable change” and “change difference” anchor-based methods. These results can be used in estimates for sample size calculation and as a reference for regulators.

A 1% deviation has been considered the cut-off that maximized sensitivity and specificity to detect objective lameness and orthopedic disease [5]. This value is in line with our MCID estimate. However, a deviation of two has been shown to maximize the combined sensitivity and specificity for objective lameness and orthopedic disease alone [5]. Future studies should evaluate the possibility that selecting more stringent deviation values may increase the odds of detecting significant changes due to treatment. Some considerations are also required for SI. One is that a naturally present low level of asymmetry is observed even in healthy animals [37,38]. Also, a recent report described some overlap in SI of healthy and dogs with hip OA. Therefore, the authors recommended against using this method for diagnostic purposes [39]. Nevertheless, our results show significant differences between the groups, indicating that weight-bearing SI can be used to monitor and assess response to treatment in OA patients.

A point can be made for combining the two measurements and collecting information on all four limbs [28]. Dogs can show complex compensation mechanisms in the presence of orthopedic disease, and load redistribution can occur from side to side or pelvic to thoracic [40,41]. Still, the calculation of symmetry indexes is commonly performed in quadruped animals [29,42,43]. Results from the weight-bearing evaluation of the animals in this study show that the major compensation occurs in the contralateral thoracic limb rather than side-to-side [14]. It was also noted that some animals with the same hip OA grade would show side-to-side compensation. In contrast, others would exhibit pelvic-to-thoracic compensation [14]. This emphasizes the importance of evaluation as individual animals instead of as groups, thus helping the clinician in their decision making [19].

This study had some limitations. The data were obtained from a specific population of dogs, and future studies should focus on a broader sample to include different breeds and body conformations. Additionally, animals with OA from other joints should be included. Also, it is important to define what constitutes a client-acceptable clinical state. This threshold can be defined as the point where a client is likely to define the outcome as “satisfactory”. The substantial clinical benefit level, i.e., the clinical value that the client considers “substantial improvement”, also needs to be determined [28].

## 5. Conclusions

In this study, we presented estimates of MCIDs for deviation and SI for dogs with hip OA, evaluated with a weight bearing evaluation platform. The MCDIs were set at −1 for deviation and −10 for SI. These estimates can be used for research and patient monitoring. Future studies should look at evaluating MCDIs in animals with OA in other joints and animals from a different clinical context.

## Figures and Tables

**Table 1:** Mean and standard deviation (SD) pre-treatment and 15 days after-treatment scores for deviation and symmetry index. * indicates significance.

Measurement	*n*	T0		+15 d		*p* Value
		Mean	SD	Mean	SD	
Deviation	160	3.5	3.0	1.7	1.6	<0.01 *
Symmetry Index	160	28.1	27.5	13.9	13.1	<0.01 *

**Table 2:** Scores in the “the same” and “somewhat better” groups at T0 and +15 d. * indicates significance.

Measurement	“The Same” Group		“Somewhat Better” Group		*p* Value
	Mean	SD	Mean	SD	
Deviation					
*n*	100		60		
T0	3.0	2.8	5.0	3.4	0.593
15 d	1.6	1.4	1.9	1.9	0.335
mean change	−1.4		−3.1		<0.01 *
Symmetry Index					
*n*	100		60		
T0	25.0	27.5	37.8	25.9	0.619
15 d	13.9	12.7	13.9	14.5	0.295
mean change	−11.1		−23.9		<0.01 *

**Table 3:** MCIDs for deviation and symmetry index. Legend: AC—average change; AUC—area under the curve; CD—change difference; MDC—minimum detectable change; ROC—receiver operating characteristic; SEM—standard error of measurement.

Measurement	Anchor-Based				Distribution-Based	
	AC	CD	MDC	ROC Curve (AUC)	Effect Size	SEM
Deviation	−3.1	−0.3	−1.6	−1.5 (0.760)	±0.29	±0.16
Symmetry Index	−23.9	−10.0	−14.0	−11.4 (0.729)	±2.88	±1.31

## Data Availability

All data generated or analyzed during this study are included in this published article.

## References

[1] Bliss S., Zink C., Van Dyke J. (2018). Musculoskeletal Structure and Physiology. Canine Sports Medicine and Rehabilitation.

[2] Anderson K.L., O’Neill D.G., Brodbelt D.C., Church D.B., Meeson R.L., Sargan D., Summers J.F., Zulch H., Collins L.M. (2018). Prevalence, duration and risk factors for appendicular osteoarthritis in a UK dog population under primary veterinary care. Sci. Rep..

[3] Enomoto M., Castro N., Hash J., Nakanishi A., Taylor A., Snyder A., Ross-Estrada M., Ferris K., Deresienski D., Frey E. (2022). Prevalence of radiographic osteoarthritis and associated clinical signs in young dogs. NC State College of Veterinary Medicine Research Forum.

[4] Alves J.C., Santos A., Jorge P., Lavrador C., Carreira L.M. (2021). Comparison of clinical and radiographic signs of hip osteoarthritis in contralateral hip joints of fifty working dogs. PLoS ONE.

[5] Clough W.T., Canapp S., Taboada L., Dycus D.L., Leasure C.S. (2018). Sensitivity and specificity of a weight distribution platform for the detection of objective lameness and orthopaedic disease. Vet. Comp. Orthop. Traumatol..

[6] Meijer E., Bertholle C.P., Oosterlinck M., van der Staay F.J., Back W., van Nes A. (2014). Pressure mat analysis of the longitudinal development of pig locomotion in growing pigs after weaning. BMC Vet. Res..

[7] Wanstrath A.W., Hettlich B.F., Su L., Smith A., Zekas L.J., Allen M.J., Bertone A.L. (2016). Evaluation of a single intra-articular injection of autologous protein solution for treatment of osteoarthritis in a canine population. Vet. Surg..

[8] Lascelles B.D.X., Roe S.C., Smith E., Reynolds L., Markham J., Marcellin-Little D., Bergh M.S., Budsberg S.C. (2006). Evaluation of a pressure walkway system for measurement of vertical limb forces in clinically normal dogs. Am. J. Vet. Res..

[9] Mölsä S.H., Junnila J.T., Laitinen-Vapaavuori O.M., Hielm-Björkman A.K., Hyytiäinen H.K. (2012). Use of bathroom scales in measuring asymmetry of hindlimb static weight bearing in dogs with osteoarthritis. Vet. Comp. Orthop. Traumatol..

[10] Seibert R., Marcellin-Little D.J., Roe S.C., DePuy V., Lascelles B.D.X. (2012). Comparison of Body Weight Distribution, Peak Vertical Force, and Vertical Impulse as Measures of Hip Joint Pain and Efficacy of Total Hip Replacement. Vet. Surg..

[11] Clough W.T., Canapp S. (2018). Assessing clinical relevance of weight distribution as measured on a stance analyzer through comparison with lameness determined on a pressure sensitive walkway and clinical diagnosis. Vet. Comp. Orthop. Traumatol..

[12] Bosscher G., Tomas A., Roe S.C., Marcellin-Little D.J., Lascelles B.D.X. (2017). Repeatability and accuracy testing of a weight distribution platform and comparison to a pressure sensitive walkway to assess static weight distribution. Vet. Comp. Orthop. Traumatol..

[13] Wilson M.L., Roush J.K., Renberg W.C. (2019). Single-day and multiday repeatability of stance analysis results for dogs with hind limb lameness. Am. J. Vet. Res..

[14] Alves J., Santos A., Jorge P., Lavrador C., Carreira L.M. (2022). Characterization of Weight-bearing Compensation in Dogs with Bilateral Hip Osteoarthritis. Top. Companion Anim. Med..

[15] Phelps H.A., Ramos V., Shires P.K., Werre S.R. (2007). The effect of measurement method on static weight distribution to all legs in dogs using the Quadruped Biofeedback System. Vet. Comp. Orthop. Traumatol..

[16] Cole G.L., Millis D. (2017). The effect of limb amputation on standing weight distribution in the remaining three limbs in dogs. Vet. Comp. Orthop. Traumatol..

[17] White D.A., Harkin K.R., Roush J.K., Renberg W.C., Biller D. (2020). Fortetropin inhibits disuse muscle atrophy in dogs after tibial plateau leveling osteotomy. PLoS ONE.

[18] Ben-Amotz R., Dycus D., Levine D., Arruda A.G., Fagan N., Marcellin-Little D. (2020). Stance and weight distribution after tibial plateau leveling osteotomy in forelimb and hind limb amputee dogs. BMC Vet. Res..

[19] Brown D.C., Bell M., Rhodes L. (2013). Power of treatment success definitions when the Canine Brief Pain Inventory is used to evaluate carprofen treatment for the control of pain and inflammation in dogs with osteoarthritis. Am. J. Vet. Res..

[20] Kvien T.K., Heiberg T., Hagen K.B. (2007). Minimal clinically important improvement/difference (MCII/MCID) and patient acceptable symptom state (PASS): What do these concepts mean?. Ann. Rheum. Dis..

[21] Alves J.C., Santos A., Jorge P. (2022). Initial psychometric evaluation of the Portuguese version of the Canine Brief Pain Inventory. Am. J. Vet. Res..

[22] Sedaghat A.R. (2019). Understanding the Minimal Clinically Important Difference (MCID) of Patient-Reported Outcome Measures. Otolaryngol.–Head Neck Surg..

[23] Lascelles B., Freire M., Roe S., DePuy V., Smith E., Marcellin-Little D. (2010). Evaluation of functional outcome after BFX total hip replacement using a pressure sensitive walkway. Vet. Surg..

[24] Alves J.C., Santos A., Jorge P., Lavrador C., Carreira L.M. (2020). A report on the use of a single intra-articular administration of autologous platelet therapy in a naturally occurring canine osteoarthritis model—A preliminary study. BMC Musculoskelet. Disord..

[25] Alves J.C., Santos A., Jorge P., Lavrador C., Carreira L.M. (2021). Intraarticular triamcinolone hexacetonide, stanozolol, Hylan G-F 20 and platelet concentrate in a naturally occurring canine osteoarthritis model. Sci. Rep..

[26] Alves J.C., Santos A., Jorge P. (2021). Platelet-rich plasma therapy in dogs with bilateral hip osteoarthritis. BMC Vet. Res..

[27] Alves J.C., Innes J.F. (2023). Minimal clinically-important differences for the “Liverpool Osteoarthritis in Dogs” (LOAD) and the “Canine Orthopedic Index” (COI) in dogs with osteoarthritis. PLoS ONE.

[28] Innes J.F., Morton M.A., Lascelles B.D.X. (2023). Minimal clinically-important differences for the ‘Liverpool Osteoarthritis in Dogs’ (LOAD) and the ‘Canine Orthopedic Index’ (COI) client-reported outcomes measures. PLoS ONE.

[29] Brown D.C., Boston R.C., Coyne J.C., Farrar J.T. (2008). Ability of the canine brief pain inventory to detect response to treatment in dogs with osteoarthritis. J. Am. Vet. Med. Assoc..

[30] Walton M.B., Cowderoy E., Lascelles D., Innes J.F. (2013). Evaluation of construct and criterion validity for the ‘Liverpool Osteoarthritis in Dogs’ (LOAD) clinical metrology instrument and comparison to two other instruments. PLoS ONE.

[31] Volstad N., Sandberg G., Robb S., Budsberg S. (2017). The evaluation of limb symmetry indices using ground reaction forces collected with one or two force plates in healthy dogs. Vet. Comp. Orthop. Traumatol..

[32] Alves J.C., Santos A., Jorge P., Lavrador C., Carreira L.M. (2020). A Pilot Study on the Efficacy of a Single Intra-Articular Administration of Triamcinolone Acetonide, Hyaluronan, and a Combination of Both for Clinical Management of Osteoarthritis in Police Working Dogs. Front. Vet. Sci..

[33] Alves J.C., Santos A., Jorge P., Carreira L.M. (2022). A randomized double-blinded controlled trial on the effects of photobiomodulation therapy in dogs with osteoarthritis. Am. J. Vet. Res..

[34] Coretti S., Ruggeri M., McNamee P. (2014). The minimum clinically important difference for EQ-5D index: A critical review. Expert Rev. Pharmacoecon. Outcomes Res..

[35] Revicki D., Hays R.D., Cella D., Sloan J. (2008). Recommended methods for determining responsiveness and minimally important differences for patient-reported outcomes. J. Clin. Epidemiol..

[36] Ogura T., Ackermann J., Barbieri Mestriner A., Merkely G., Gomoll A.H. (2018). Minimal Clinically Important Differences and Substantial Clinical Benefit in Patient-Reported Outcome Measures after Autologous Chondrocyte Implantation. CARTILAGE.

[37] Souza A.N.A., Escobar A.S.A., Germano B., Farias C.L.F., Gomes L.F.F., Matera J.M. (2019). Kinetic and Kinematic Analysis of Dogs Suffering from Hip Osteoarthritis and Healthy Dogs Across Different Physical Activities. Vet. Comp. Orthop. Traumatol..

[38] Voss K., Imhof J., Kaestner S., Montavon P.M. (2007). Force plate gait analysis at the walk and trot in dogs with low-grade hindlimb lameness. Vet. Comp. Orthop. Traumatol..

[39] Brønniche Møller Nielsen M., Pedersen T., Mouritzen A., Vitger A.D., Nielsen L.N., Poulsen H.H., Miles J.E. (2020). Kinetic gait analysis in healthy dogs and dogs with osteoarthritis: An evaluation of precision and overlap performance of a pressure-sensitive walkway and the use of symmetry indices. PLoS ONE.

[40] Kennedy S., Lee D.V., Bertram J.E.A., Lust G., Williams A.J., Soderholm L.V., Hamilton S., Bliss S.P., Dykes N.L., Todhunter R.J. (2003). Gait evaluation in hip osteoarthritic and normal dogs using a serial force plate system. Vet. Comp. Orthop. Traumatol..

[41] Vassalo F.G., Rahal S.C., Agostinho F.S., Mamprim M.J., Melchert A., Kano W.T., Mesquita L.d.R., Doiche D.P. (2015). Gait analysis in dogs with pelvic fractures treated conservatively using a pressure-sensing walkway. Acta Vet. Scand..

[42] Pashuck T.D., Kuroki K., Cook C.R., Stoker A.M., Cook J.L. (2016). Hyaluronic acid versus saline intra-articular injections for amelioration of chronic knee osteoarthritis: A canine model. J. Orthop. Res..

[43] Spadari A., Romagnoli N., Predieri P., Borghetti P., Cantoni A., Corradi A. (2013). Effects of intraarticular treatment with stanozolol on synovial membrane and cartilage in an ovine model of osteoarthritis. Res. Vet. Sci..

